# A mammary adenocarcinoma murine model suitable for the study of cancer immunoediting

**DOI:** 10.1186/1423-0127-21-52

**Published:** 2014-05-30

**Authors:** Lucas Pagura, Juan Manuel Cáceres, Albertina Cardinale, Olga Graciela Scharovsky, Ricardo José Di Masso, Mariano Federico Zacarías-Fluck, María José Rico, Viviana Rosa Rozados

**Affiliations:** 1 Instituto de Genética Experimental, Facultad de Ciencias Médicas, Universidad Nacional de Rosario, Santa Fe 3100, (2000) Rosario, Argentina; 2 Preclinical Research, Vall d’Hebron Institute of Oncology (VHIO), Barcelona, Spain; 3 CIC-UNR, Rosario, Argentina; 4 CONICET, Buenos Aires, Argentina

**Keywords:** Cancer immunoediting, Murine models, Mammary adenocarcinoma, Mathematical model

## Abstract

**Background:**

Cancer immunoediting is a dynamic process composed of three phases: elimination (EL), equilibrium (EQ) and escape (ES) that encompasses the potential host-protective and tumor-sculpting functions of the immune system throughout tumor development. Animal models are useful tools for studying diseases such as cancer. The present study was designed to characterize the interaction between mammary adenocarcinoma M-406 and CBi, CBi^−^ and CBi/L inbred mice lines.

**Results:**

The mammary adenocarcinoma M-406 developed spontaneously in a CBi mouse. CBi/L and CBi^−^ mice were artificially selected for body conformation from CBi. When CBi mice are s.c. challenged with M-406, tumor growths exponentially in 100% of animals, while in CBi^−^ the tumor growths briefly and then begins a rejection process in 100% of the animals. In CBi/L the growth of the tumor shows the three phases: 51.6% in ES, 18.5% in EQ and 29.8% in EL.

**Conclusions:**

The results obtained support the conclusion that the system M-406 plus the inbred mouse lines CBi, CBi^−^ and CBi/L, is a good murine model to study the process of tumor immunoediting.

## Background

Animal models have been in the past, are still at present, and almost surely will continue to be in the future, very useful tools in biomedical investigation [1,2]. In the field of cancer research it is widely known that both, spontaneous and chemically-induced tumors, can develop in rats and mice [3] as well as in other species [4] and that mutations identified in murine models are often similar to those observed in human tumor cells [5,6]. Although *in vivo* models of pathologic processes are useful only if they provide information that can be extrapolated to other species of interest, particularly to humans, and it is known that no model behaves ideally, it is also true that they allow not only to replicate but also, in some cases, to anticipate the genetic architecture of complex human phenotypes as is the case of cancer disease. While *in vitro* models provide a plain system for studying some of their components in controlled conditions, *in vivo* models allow obtaining answers that go beyond the cell or tissue level since they involve a whole living organism who can be studied during the evolution of disease. CBi is an inbred mouse strain derived from an outbred population generated by crossing BALB/c, Rockland, NIH and Swiss mice. It was generated to be used as a base population of broad genetic basis and as the control line of an experiment of artificial body-conformation selection which gave rise, among others, to CBi^−^ and CBi/L mice lines [7,8]. During selection, the lines were inbred by limiting the population size [9] until an average theoretical inbreeding coefficient of approximately 0.985 was reached. From then on, a regular system of inbreeding involving full-sib (FS) mating was applied and maintained for more than 30 generations giving rise to CBi/L FS, CBi^**−**^ FS and CBi FS lines. Mice belonging to these FS lines show a heterogeneous behavior in their resistance and susceptibility to parasites [10], in spontaneous mammary carcinogenesis [11] and when challenged with M-406 mammary adenocarcinoma, a tumor which arose spontaneously in a female CBi mouse. While in CBi FS the tumor grows exponentially in 100% of challenged animals, in CBi^**−**^ FS the tumor grows briefly and then begins a rejection process in 100% of the animals. In CBi/L FS mice the tumor behavior is more complex as it begins with an exponential growth pattern in all challenged mice and, after a variable length period, the tumor is completely eliminated in some individuals while in others continues growing exponentially or enters in a state of equilibrium where no additional growth is detectable. After the equilibrium phase the tumor resumes the exponential growth becoming lethal in some animals, while in others it begins a process of rejection until its complete elimination.

The interaction between tumor cells and the immune system is very complex. It is currently accepted that the immune system can not only protect against tumor development but it is also capable of stimulating tumor growth. On one side, both innate and adaptive immune mechanisms act in synergism in order to counteract tumor growth before it becomes clinically apparent. On the other side, the immune system can also promote tumor progression through chronic inflammation, immunoselection of poorly immunogenic variants and by suppressing antitumor immunity. These dual host-protective and tumor-promoting actions of immunity are referred to as cancer immunoediting [12]. This phenomenon consists of three steps: elimination, equilibrium and escape, which are known as the three E´s of tumor immunoediting [12-14]. The present study was designed to characterize the interaction between mammary adenocarcinoma M-406 and CBi FS, CBi^**−**^ FS and CBi/L FS inbred mice lines, as a model for studying the process of cancer immunoediting.

## Methods

### Animals

Ten-weeks-old CBi FS, CBi^**−**^ FS and CBi/L FS (from here on CBi, CBi^**−**^ and CBi/L, respectively) female mice belonging to the Institute of Experimental Genetics, School of Medical Sciences, National University of Rosario breeding facilities, were used. All mice were kept in the same room under the same breeding conditions (23 ± 1°C, on a 12-hour-on/12-hour-off light cycle) and received the same diet (Cargill Laboratory Chow, pelletized) and water *ad libitum*. Animals were treated in accordance with the institutional regulations which comply with the guidelines issued by the Canadian Council on Animal Care [15].

### Tumor

According to Squartini’s classification [16] M-406 is a type B semi-differentiated mammary adenocarcinoma. It is a triple-negative tumor (ER^−^, PR^−^, HER-2^−^) that arose spontaneously in an inbred CBi female mouse and it is maintained *in vivo* by serial intraperitoneal passages in syngeneic mice.

### Experimental model

All experiments were performed with prior approval from the Bioethics Committe of the Faculty of Medical Sciences of the UNR, Argentine, IMED 249, Res N°1383/12.

#### Tumor growth with inoculum by trocar

Female CBi (n = 52), CBi^**−**^ (n = 52) and CBi/L (n = 150) mice were s.c. challenged in the lateral flank with M-406 by trocar (three tumor fragments ≈ 8x10^5^ cells) on day 0. Minor and major tumor diameters were measured with a caliper three times a week from day 3 on, and tumor volume was estimated according to the formula [V = (minor diameter)^2^ × major diameter × 0.4]. Tumor volume versus time elapsed since tumor challenge data were adjusted with an exponential model [Vt = Start.e^(k.t)], where Start is the value of tumor volume (mm^3^) at t = 0, Vt is the tumor volume at t time, t indicates days post tumor inoculation and k is the exponential growth rate (volume being added to the system proportional to the volume already present). Tumor volume doubling time (TvDT) was calculated as TvDT = ln2/k. Animals were classified as, Escape (ES): animals in which the tumor grew exponentially, Equilibrium (EQ): animals in which tumor volume remained constant for at least 10 consecutive days, and Elimination (EL): animals in which the tumor was completely eliminated.

#### Evaluation of spontaneous metastasis

When tumors in ES phase (CBi and CBi/L lines) reached the maximum volume ethically permitted by the Canadian Council on Animal Care [15], mice were euthanized, their lungs were excised and fixed in Bouin solution and the metastatic foci were determined. The lungs are the main metastatic site of M-406 tumor when growing s.c. in CBi and CBi/L lines.

#### Tumor growth with counted inoculum

In a subsequent experiment CBi and CBi^**−**^ mice (n = 5/group/line) were challenged with M-406 in the lateral flank according to the following specifications: Group I with trocar (three tumor fragments ≈ 8×10^5^ cells) and Groups II, III and IV with suspensions containing 8×10^5^, 4×10^5^ and 2×10^5^ tumor cells for CBi and 8×10^5^, 16×10^5^ and 32×10^5^ tumor cells for CBi^**−**^, respectively.

In both experiments, the behavior of tumor bearing animals was monitored daily and when the tumor reached the maximum volume allowed by ethical standards, mice were euthanized by CO_2_ overexposure.

### Cell suspensions

*Spleens cells*: CBi^**−**^ and CBi/L animals, bearing tumors in EL phase, were euthanized by CO_2_ overexposure, their spleens were excised and cellular suspensions were obtained by mechanical disruption in RPMI medium.

*Tumor cells*: tumors from CBi mice bearing i.p. M-406 were excised and cell suspensions were obtained by mechanical disruption in RPMI medium.

### Winn assay

**Figure 1:**
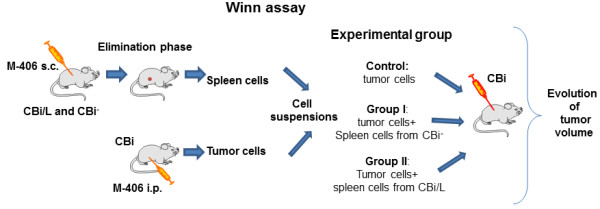
Experimental model of Winn assay.

CBi animals (n = 10/group) were inoculated s.c. in the right flank with 0.1 ml of different cell suspensions containing: 410^5^ tumor cells in RPMI medium (Control), 4×10^5^ tumor cells + 7×10^5^ spleen cells from CBi^**−**^ EL (Group I) and 4×10^5^ tumor cells + 7×10^5^ spleen cells from CBi/L EL (Group II). Tumor size was monitored three times a week until tumor size reached ethical limits (Figure 1).

### Conditioned medium

*Tumor conditioned medium (CT)*: tumor cells obtained from CBi mice bearing i.p. M-406 were cultured in RMPI + 10% FBS for 24 h at 37°C and 5% CO_2_ (4×10^5^ cells/100 μl). The suspension was centrifuged 10 min at 2000 rpm and the supernatant was separated and stored at -20°C.

*Mononuclear cells (MO) conditioned medium (CMO)*: blood was drawn by cardiac puncture with EDTA from mice belonging to the three lines: *Naïve* (N): non-challenged with tumor (CMO-CBi N, CMO-CBi^**−**^ N and CMO-CBi/L N) and M-406 bearers (CMO-CBi ES, CMO-CBi^**−**^ EL, CMO-CBi/L EL and CMO-CBi/L ES). Mononuclear cells were obtained by Ficoll-Paque PLUS (GE Healthcare, USA) gradient centrifugation. Then, 4×10^5^ mononuclear cells/100 μl were cultured in RPMI + 10% FBS for 24 h at 37°C and 5% CO_2_. The suspension was centrifuged 10 min at 2000 rpm and the supernatant was separated and stored at −20°C.

### Proliferation assay

*Tumor cells*: M-406 (5×10^4^ cells) was cultured with different CMO (100 μl) and with RPMI (Control group, reference value, 100%).

*Mononuclear cells*: MO (5×10^4^ cells) from *naïve* CBi (MO-CBi N), CBi^**−**^ (MO-CBi^**−**^ N) and CBi/L (MO-CBi/L N) mice were cultured with CT (100 μl).

**Figure 2:**
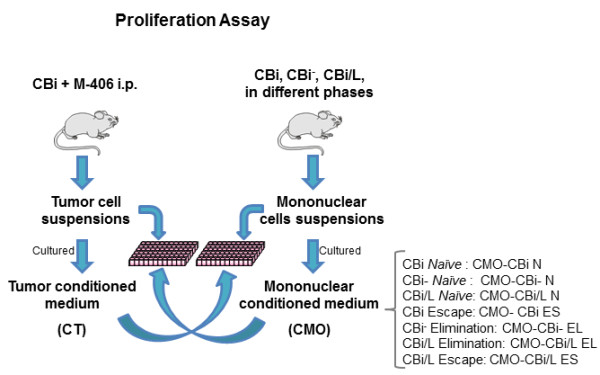
Experimental model of proliferation assay.

Cells were incubated for 24 h, at 37°C and 5% of CO_2_ and cell proliferation was evaluated with WST-1 kit (Roche, Argentina) (Figure 2).

### Statistical analysis

Statistically significant differences between groups (P values less than 0.05) were assessed using Mann–Whitney *U* test or Kruskal-Wallis one-way analysis of variance by ranks followed by Dunn's post-test, Student’s *t* test, Log-rank-Mantel-Cox test, one way ANOVA followed by Tukey´s multiple comparison test and Spearman correlation, as appropriate (GraphPad, version 3.0).

## Results

### Tumor growth with inoculum by trocar

**Figure 3:**
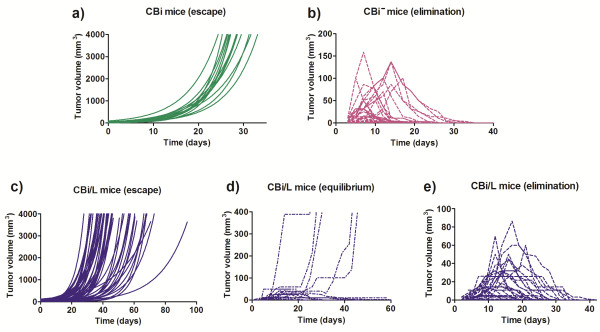
**Evolution of M-406 tumor growth with inoculum by trocar in the three lines of mice. a)** CBi (ES); **b)** CBi^**−**^ (EL); **c)** CBi/L (ES); **d)** CBi/L (EQ); **e)** CBi/L (EL). Escape curves were fitted to an exponential growth equation.

Irrespective of the mouse line, M-406 grew in all challenged animals. In CBi mice (Figure 3a) the tumor maintained an exponentially growing pattern (escape phase, ES) until it reached the maximum volume allowed according to ethical guidelines. On the contrary, after a short period of initial growth all CBi^**−**^ mice rejected the tumor (elimination phase, EL) (Figure 3b). In CBi/L the tumor displayed the three phases of cancer immunoediting as it was eliminated (EL) in 30.0% of challenged mice, reached an equilibrium phase (EQ) in 18.7% and maintained a continuous growing pattern (ES) in the remainder 51.3% (Figure 3e,c and d, respectively) (Table 1).

**Table 1:** Animals in escape, equilibrium and elimination phases

	**CBi**	**CBi**^**−**^	**CBi/L**
**Take**	100%	100%	100%
	(52/52)	(52/52)	(150/150)
**Escape**	100%	-	51,3%
	(52/52)		(77/150)
**Equilibrium**	-	-	18,7%
			(28/150)
**Elimination**	-	100%	30,0%
		(52/52)	(45/150)

**Figure 4:**
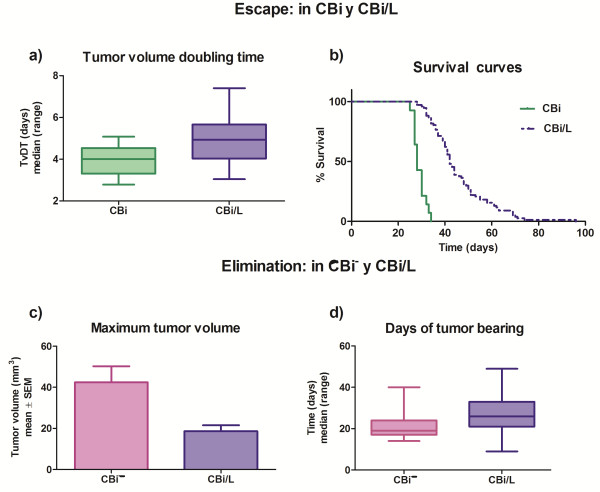
**Escape and elimination phases in CBi, CBi**^**− **^**and CBi/L mice. a)** Tumor volume doubling time (TvDT); CBi *vs* CBi/L (P = 0.0014, Mann Whitney test); **b)** Survival curves of CBi and CBi/L mice in ES phase (P < 0.0001, Log-rank test Mantel-Cox); **c)** Maximun tumor volume: CBi^**−**^*vs* CBi/L (P = 0.0023, Student’s *t* test); **d)** Days of tumor bearing: CBi^**−**^*vs* CBi/L (P = 0.0019, Mann Whitney test).

*Escape phase*: it was only observed in CBi and CBi/L mice. In CBi mice, TvDT (median; range) [4.00 (2.78-5.08)] was lower (P = 0.0014) than that of CBi/L animals, [4.94 (3.04-7.40)] (Figure 4a). These differences were mirrored in the survival behavior as median survival times of 28 and 42 days were observed in CBi and CBi/L mice, respectively (P < 0.0001) (Figure 4b).

*Elimination phase*: the maximum tumor volume reached before the beginning of rejection was significantly higher (P = 0.023) in CBi^**−**^ mice (mean ± SEM: 42.5 ± 7.73 mm^3^) than in CBi/L mice (18.7 ± 2.86 mm^3^) (Figure 4c). However, tumor-bearing time was significantly lower (P = 0.0019) in CBi^**−**^ mice (days: median; range) [19 (14–40)] than in CBi/L mice [26 (9–49)], indicating that the actual elimination of the tumor is faster in CBi^**−**^ than in CBi/L (Figure 4d).

*Equilibrium phase*: it was only observed in CBi/L animals which tumor volumes were stable over a period of at least 10 days. The average tumor volume was 37.6 ± 14.70 mm^3^.

In all mice lines the presence of tumor was studied by classic H&E techniques (data not shown).

### Evaluation of spontaneous metastasis

**Figure 5:**
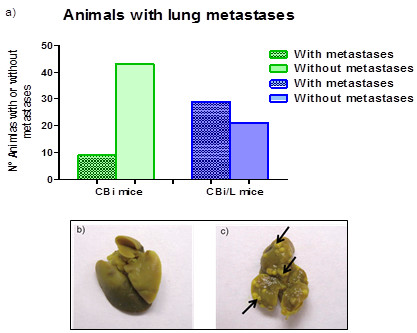
**Lung metastasis development in CBi (n = 52) and CBi/L (n = 50) mice. a)** Percentage of mice developing metastasis. CBi vs CBi/L (P = 0.0001, Fisher's Exact Test); **b)** Lung without metastasis; **c)** Lung with metastasis.

The number of animals with lung metastases foci was lower in CBi line [17% (9/52)] than in CBi/L [58% (29/50)] (P < 0.0001), (Figure 5a). The appearance of lungs without and with metastases is shown in Figures 5b and c, respectively.

### Tumor growth with counted inoculum

**Figure 6:**
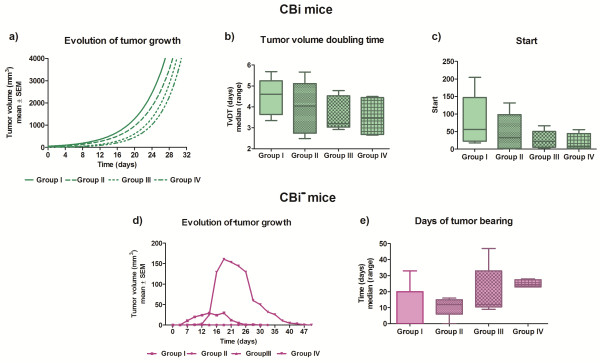
**Evolution of M-406 tumor growth with counted inoculum in CBi and CBi**^**− **^**mice.** Group I: tumor challenge with trocar and Groups II, III and IV, inoculated with tumor cells suspension: 8x10^5^, 16x10^5^ and 32x10^5^ cells, respectively. **a)** Tumor volume in CBi mice; **b)** TvDT in CBi mice; **c)** Start value in CBi mice; **d)** Tumor volume in CBi^**−**^ mice, **e)** Days of tumor bearing in CBi^−^ mice.

M-406 grew in all CBi mice groups (ES) until it reached the maximum ethically allowed volume (Figure 6a). At day 30 post-challenge, tumor volume showed the following decreasing pattern (GI > GII > GIII > GIV) compatible with the growing size of the inoculum although no significant differences among groups were evident either on TvDT (Figure 6b) or in Start values (START) (Figure 6c).

In CBi^**−**^ mice the four groups showed 100% of tumor takes and 100% of tumor regression (Figure 6d). Tumor bearing time tend to be longer in GIV (47 days) in comparison to GI (28), GII (30) and GIII [16], without reaching statistical significance (Figure 6e).

### Winn assay

**Figure 7:**
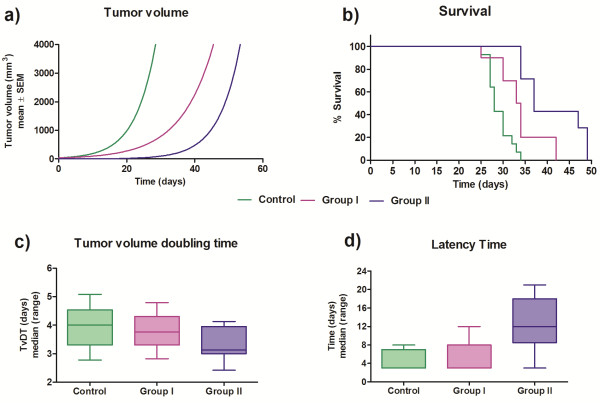
**Winn assay.** Control: 4x10^5^ tumor cells in RPMI medium; Group I: 4x10^5^ tumor cells + 7x10^5^ spleen cells from CBi^**−**^ EL; Group II: 4x10^5^ tumor cells + 7x10^5^ spleen cells from CBi/L EL. **a)** Curves were fitted to an exponential growth equation (P < 0,0001); **b)** Survival proportions (P = 0.001, Log-rank Mantel-Cox test); **c)** TvDT (N.S. Kruskal-Wallis test); **d)** Latency time (P = 0.002, Kruskal-Wallis test).

The exponential growth pattern of tumor volumes differed among groups (P < 0.0001) (Figure 7a). The co-inoculation of tumor cells plus spleen cells from both CBi^**−**^ and CBi/L in elimination phase (Groups I and II, respectively) had a negative effect on tumor growth. As a consequence, survival curves also showed significant differences. The control group showed a median survival time of 28 days, whereas groups I and II had a significantly higher median survival time: 33.5 days and 37 days, respectively (P = 0.001) (Figure 7b). Although there were no significant differences in TvDT among groups (P > 0.05) (Figure 7c), tumor latency, defined as the time elapsed from the inoculation of the tumor cells until the tumor became palpable, was significantly lower in animals of the Control group (days: median; range [3 [3-8]]) and Group I [3 [3-12]] than those of Group II [12 [3-21]] (P = 0.002) (Figure 7d).

### Effect of mononuclear cells’ conditioned media on tumor proliferation

**Figure 8:**
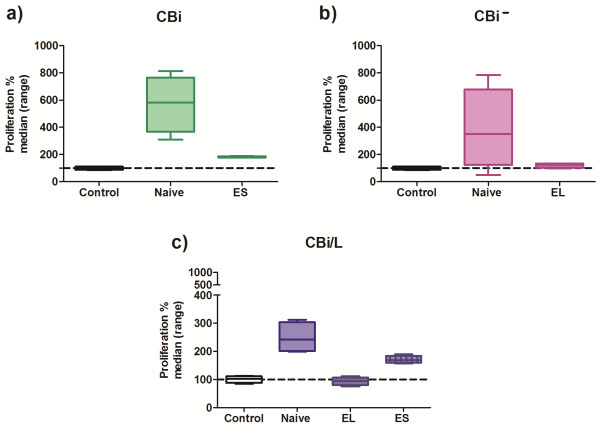
**Percentage of proliferation of M-406 cells incubated with mononuclear cells conditioned medium (n = 4): a) CBi: *Naïve vs *** **Control, P = 0.0073; b) CBi−, N.S.; c) CBi/L: *Naïve vs *** **Control and vs EL, P = 0.0084; (Kruskal-Wallis test and Dunn´s multiple comparisons test).**

The incubation of M-406 cells with CMO-CBi N increased the proliferation with respect to Control group (P = 0.0073). No differences were observed among the other groups (CMO-CBi N and Control vs CMO-CBi ES) (Figure 8a). No differences with Control group were observed in M-406 cells proliferation when incubated with CMO-CBi^**−**^ N or CMO-CBi^**−**^ EL (Figure 8b). On the contrary, when tumor cells were incubated with CMO-CBi/L N the proliferation was significantly higher than that obtained with cells incubated with RPMI (Control) or CMO-CBi/L EL (P = 0.0084) (Figure 8c).

### Effect of tumor conditioned media (CT) on mononuclear cells’ proliferation

**Figure 9:**
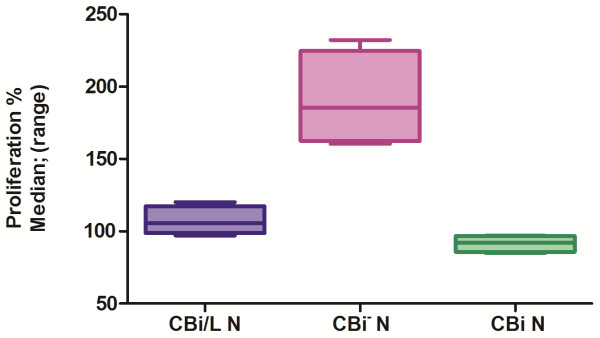
**Percentage of proliferation of mononuclear cells from Naïve mice incubated with conditioned media from M-406 tumor cells (CT).** MO-CBi^−^ N vs MO-CBi N and vs MO-CBi/L N P = 0.0073 (Kruskal-Wallis test).

When proliferation of mononuclear cells from *Naïve* CBi, CBi^**−**^ or CBi/L mice incubated with CT was evaluated, MO-CBi^**−**^ N proliferation was higher than MO-CBi N and MO-CBi/L N (P = 0.0073) (Figure 9).

## Discussion

In the last fifteen years, the interest in cancer immunosurveillance has re-emerged. Shankaran *et al*. have shown that, besides its protective role against disease, the immune system may also promote the emergence of primary tumors with reduced immunogenicity that are capable of escaping immune recognition and destruction [13]. These findings prompted the development of the cancer immunoediting hypothesis to encompass more broadly the potential host-protective and tumor-sculpting functions of the immune system throughout tumor development [14]. Cancer immunoediting which is known as the three E’s theory, is a dynamic process composed of three phases: ES, EQ and EL. The *Elimination phase* corresponds to the original concept of cancer immunosurveillance whereby cancer cells are successfully recognized and destroyed by the immune system. If tumor cells are not completely eliminated they may proceed into an *Equilibruim phase* in which the immune system is able to control tumor growth but is incapable to eliminate it completely. Over the time, however, cancer may overcome the entire immune response and enter into the third phase, or *Escape phase*, during which it progresses and metastasizes [17].

The inbred mouse lines CBi, CBi^**−**^ and CBi/L of the Institute of Experimental Genetics, School of Medical Sciences, UNR, jointly with M-406 mammary adenocarcinoma, provide a suitable model for studying the three “E’s” of tumor immunoediting. Direct and correlated responses to selective pressure together with the non-directional effects of inbreeding and genetic drift suggest that these lines have fixed different allelic combinations, which would explain the uniform behavior observed within lines as well as the differences observed between them. While CBi behaves as a susceptible line in terms of mammary adenocarcinoma M-406 growth, CBi^**−**^ does it as a resistant one. Both behaviors -susceptibility or resistance- proved to be independent of the integrity and the size (number of cells) of the inoculum used. On one hand the results showed that inoculum size was only related to tumor bearing time. On the other hand, despite having quadrupled the number of tumor cells used to challenge animals with trocar, CBi^**−**^ mice were able to inhibit M-406 growth, thus confirming the status of line resistant to the challenge with this particular type of tumor. In contrast, CBi mice, independently of the number of tumor cells inoculated, even when that number was four times lower, were unable to halt M-406 growth, a fact that supports the status of susceptible line. The heterogeneous behavior observed in CBi/L could be explained if the relationship between M-406 and the immune system was modeled as a threshold character. Undoubtedly, from a genetic point of view, the immune response against a tumor is a quantitative trait as it involves many genes. According to the proposed threshold model, the immune response against tumor challenge is inherited as a continuous trait with a threshold which imposes a discontinuity on the phenotypic expression (resistance or susceptibility) of the character. Several loci in mouse, like Mmtg1, Mmtg2, Mmtg3, Mtes1, Apmt1, Apmt2 were involved in cancer-susceptibility [18]. Also, three QTLS that control tumor incidence and/or latency were mapped in the crosses between BALB/c mice (predisposed to develop spontaneous mammary tumors, especially when carrying a single normal Tp53 allele) and C57BL/6 mice (which are resistant to mammary cancer). In the aforementioned model, Dmbt1 is a solid candidate for a putative tumor suppressor gene involved in immune defense [19,20]. The genetic architecture of the trait, determines, i.e., that the allelic frequencies of those genes involved in the immune response corresponding to CBi^**−**^ and CBi mice, clearly lie above and below, respectively, of the threshold, thus expressing in each case, the resistance or susceptibility phenotypes. In contrast, gene arrangement in CBi/L localized this particular line in the boundaries of the threshold allowing the emergence of the different patterns associated with the three E’s theory [12,14].

Tumor doubling time values and survival curves indicate that the EL and ES phases differ in CBi/L line from those observed in CBi and CBi^**−**^ mice. Interestingly, both processes lasted more in CBi/L than in CBi and CBi^**−**^. In the ES phase, tumor-growth rate was slower in CBi/L than in CBi, a fact evident when tumor doubling times and survival curves were compared. At the same time, the elimination process was slower in CBi/L than in CBi^**−**^. The interaction between tumor cells and the immune response in CBi/L would be weaker than that generated in CBi^**−**^ line, but would still be effective in some cases for a complete tumor rejection. These patterns also agree with the observation that M-406 reached the maximum volume in CBi/L later than in CBi mice. The usefulness of the exponential model to characterize tumor growth from a quantitative point of view, allows using it as a tool for prognostic purposes and for measuring the therapeutic effects of different treatment modalities [21,22].

The development of metastasis is an important hurdle for cancer treatment. Interestingly, CBi/L mice showed more lung metastasis than CBi mice. The capacity to metastasize is due to factors both extrinsic and intrinsic to the tumor cells [23]. The differences observed in the development of metastasis between CBi and CBi/L could be associated with extrinsic factors like host susceptibility, immune response, neo-angiogenesis and the peritumoral stroma. Hence, in order to understand the aforementioned differences, these and other potentially involved factors should be studied. For example, having in mind the role of the intrinsic factors, it could be hypothesized that the probability of metastasis development would be higher in CBi/L than in CBi mice, because of the longer time needed by the tumor to reach the maximum allowed volume in CBi/L due to the special interaction between the tumor cells and the immune response of each host.

Evidence derived from the Winn assay indicated that spleen cells from immunized animals were able to inhibit tumor growth, at least temporarily. Differences in tumor growth rate and survival curves were evident when mice were challenged jointly with tumor cells and spleen cells derived from immunized animals. When tumor growth was studied in CBi animals challenged with M-406 cells plus spleen cells from CBi^**−**^ or CBi/L tumor-bearing mice in EL phase, different responses were observed in latency time. Although the tumor grew exponentially in all animals, the increase in latency time was evident in mice challenged with tumor cells plus CBi/L spleen cells in EL (Group II) with respect to the Control group that showed the lowest values. The response in Group I was intermediate between that of Control and Group II. These results do not agree with previous ones showing that the process of EL lasted more in CBi/L than in CBi^−^ mice (Figure 2d); so, it could be expected that MO from CBi/L would be less able to delay the beginning of tumor growth than MO from CBi^−^ mice. Such a discrepancy deserves further studies to unveil its biologic base.

In order to deepen the knowledge of CBi/M-406 model, the tumor cell growth in different CMO from naive and tumor bearing animals in the different tumor-growth phases was evaluated. The higher proliferation of M-406 cells observed when cultured with CMO from both *Naïve* CBi/L and CBi mice indicates that these media would contain factor/s like IL-2, IL-4, INF-γ, IL-10, TNF-α and many others, yet to be identified, capable to stimulate or inhibit tumor growth. This observation agrees with the results obtained by Franca [24] who found that direct contact between fresh human mononuclear cells and conditioned media from tumor cells induces the secretion of TNF-α and VEGF, factors which are clearly involved in tumor growth.

## Conclusions

The results herein described, taken together, suggest that these different inbred lines of mice plus the M-406 mammary adenocarcinoma perform as a very good model for studying the process of tumor immunoediting. Particularly, the present studies indicate that CBi/L mice, despite having a high coefficient of inbreeding, can develop any of the three phases of tumor immunoediting after being challenged with M-406 mammary adenocarcinoma, unlike CBi^**−**^ or CBi mice, which present a homogeneous behavior. The recognition of the mechanisms involved in the different phases of tumor growth, would likely lead to design different strategies for breast cancer treatment or prevention.

## Abbreviations

ER: Estrogen receptor; PR: Progesterone receptor; HER-2: Human epidermal growth factor receptor 2; FS: Full sib; TvDT: Tumor volume doubling time; ES: Escape phase; EQ: Equilibrium phase; EL: Elimination phase; CT: Tumor conditioned medium; MO: Mononuclear cells; N: *Naïve*; MO-CBi N: Mononuclear cells from *Naïve* CBi line; MO-CBi^−^ N: Mononuclear cells from *Naïve* CBi^−^ line; MO-CBi/L N: Mononuclear cells from *Naïve* CBi/L line; CMO: Conditioned medium from mononuclear cells; CMO-CBi N: Conditioned medium from mononuclear cells from *Naïve* CBi line; CMO-CBi^−^ N: Conditioned medium from mononuclear cells from *Naïve* CBi^−^ line; CMO-CBi/L N: Conditioned medium from mononuclear cells from *Naïve* CBi/L line; CMO-CBi ES: Conditioned medium from mononuclear cells from *Naïve* CBi line in Escape Phase; CMO-CBi^−^ EL: Conditioned medium from mononuclear cells from *Naïve* CBi^−^ line in Elimination Phase; CMO-CBi/L EL: Conditioned medium from mononuclear cells from *Naïve* CBi/L line in Elimination Phase; CMO-CBi/L ES: Conditioned medium from mononuclear cells from *Naïve* CBi/L line in Escape Phase.

## Competing interests

The authors declare that they have no competing interests.

## Authors’ contributions

LP carried out all the experiments, performed the statistical analysis and participated in the writing of the manuscript, JMC and AC participated in many of the experiments. MJR and VRR conceived of the study, and participated in its design and coordination and helped to draft the manuscript. RJDM performed a body-conformation selection which gave rise, among others, to CBi- and CBi/L mice lines, participated in the experimental design, performed the statistical analysis and helped to draft the manuscript. MFZF and OGS participated in the experimental design and help to draft the manuscript.

## Authors’ information

Viviana Rosa Rozados and María José Rico are senior authors.
